# Is there evidence that playing games promotes social skills training for autistic children and youth?

**DOI:** 10.1177/13623613241277309

**Published:** 2024-09-21

**Authors:** Orla Walsh, Conor Linehan, Christian Ryan

**Affiliations:** University College Cork, Ireland

**Keywords:** adolescents, autism spectrum disorder, children, social communication, social skills games, systematic review, young adults, youth

## Abstract

There is increasing interest in the use of games to scaffold social skills training for autistic children and youth. However, there is no consensus on how to best approach their design, so that learning opportunities are maximised. This article presents a systematic scoping review of empirical studies that use games as social skills training for autistic children and youth. We set out to describe the different types of game-based interventions observed, to identify those that typically bring successful outcomes (while noting the social skills targeted in each), and to highlight the psychological intervention approach taken. Through a search of five databases, 3070 studies were identified and reduced, through screening, to a sample of 17 studies. Each study reported that their game successfully promoted social skills training for autistic people. Although not a part of the criteria, all studies included a technological element. However, across the sample, there was also a lack of specificity in how ‘social skills’ were defined and in which skills were chosen as the focus of the intervention. Moving forward, we recommend a more theoretically driven approach to defining behavioral targets for game-based interventions, as well as the inclusion of autistic voices earlier and more centrally in the design processes.

**Lay Abstract:**

There is growing interest in using games to help autistic children and youth learn social skills. However, there is no clear agreement on the best way to design these games to ensure they are most effective. In our research, we reviewed studies that used games to teach social skills to autistic children and youth. We aimed to describe the different types of games, identify which ones were most successful and understand the psychological methods used. We searched five databases and found 3070 studies, which we narrowed down to 17 that met our criteria. Each of these 17 studies reported that their game helped improve social skills in autistic children. Interestingly, all these studies used some form of technology, even though this was not a requirement. However, we noticed that many studies were not clear on what specific social skills they were targeting or how they defined ‘social skills’. For future work, we suggest that game-based interventions should be more clearly based on established theories. In addition, it is important to involve autistic people in the design of these games to ensure they meet their needs effectively.

## Introduction

Globally, it is estimated that roughly 1-in-100 children are autistic though some studies report higher figures (World Health Organization, 2023; Zeidan et al., 2022). As defined by the Diagnostic and Statistical Manual of Mental Disorders, Fifth Edition, autism is characterised by qualitative impairment in social communication, engagement in rituals and routines, and sensory sensitivities (American Psychiatric Association (APA), 2013). These challenges often involve difficulties in social cognition, language, communication and cognitive flexibility – the ability to adapt thinking or behaviour in response to changing demands (Charman, 2003; Ke & Moon, 2018; Lage et al., 2024).

Social skills, which integrate behavioural, cognitive and affective components, are essential for adapting to social contexts and encompass both verbal and nonverbal behaviours crucial for interpersonal communication (Giusti et al., 2011; Rao et al., 2007). Consequently, autistic people often face challenges in social competence, such as understanding social cues or navigating social interactions (Kylliäinen et al., 2020). Specific aspects of social skills, including joint attention, conversational abilities and social participation, have been the focus of research (Colombi et al., 2009; Ke & Moon, 2018). For instance, joint attention is defined as the ability to jointly focus on an object or area with another person (Salo et al., 2018).

These social challenges underscore the importance of developing cognitive flexibility and social skills. Research indicates that autistic children, even when integrated into mainstream classrooms, often report greater loneliness and poorer friendship quality compared to their peers and are more susceptible to bullying victimisation (Libster et al., 2022; Locke et al., 2010). Gender differences show autistic girls tend to stay close to peers during free play, while boys often engage in solitary activities (Dean et al., 2017). In addition, there is a significant relationship between joint engagement and loneliness, suggesting that social group participation may sometimes exacerbate feelings of isolation (Dean et al., 2023). Autistic children often prefer to have a few close friends whom they trust (Lam et al., 2020). Moreover, differences in social skills can be a predictor of social anxiety in young autistic people (Bellini, 2006), potentially hindering their ability to interact effectively with peers (Bauminger, 2006). Considering these findings, supporting autistic youth in developing their social skills through play is worthwhile.

### Intervention approaches

Social skills training (SST) is commonly used to enhance social functioning in autistic children, with group interventions particularly effective in improving social communication (Reichow et al., 2012). Group-based SST is often seen as the most efficient method (Freitag et al., 2013). Well-designed SST interventions can contribute to enhanced social and academic well-being (Foster & Bussman, 2007; Stichter et al., 2014). Various strategies have been employed in teaching social skills, with approximately a quarter of teachers reportedly using at least one evidence-based practice specific to autism (Paisley et al., 2022).

One effective SST method is video modelling, which involves demonstrating a target skill through video, using either self-modelling or peer modelling (Galligan et al., 2020). Video modelling has proven effective in promoting appropriate behaviours (Ashori & Jalil-Abkenar, 2019; Burton et al., 2013; Plavnick et al., 2015), effective communication skills (Gelbar et al., 2012), daily living skills (Aldi et al., 2016), vocational skills (Kellems & Morningstar, 2012) and reciprocal social interactions (Boudreau & Harvey, 2013).

Other intervention approaches for social skill development include community-based programmes (Koegel et al., 2012), peer mediation (Allen & Barber, 2015) and play-based interventions (Neugnot-Cerioli et al., 2015). Activities focusing on shared interests, such as mutually enjoyed activities, may facilitate connections among autistic youth (Bottema-Beutel et al., 2016; Kasari et al., 2012). For instance, LEGO been utilised as a tool to facilitate play-based social skills interventions (Legoff & Sherman, 2006). Imaginary friends also have positive impacts on understanding others and enhancing social skills (Davis, 2020; Davis et al., 2022).

School-based SST interventions are supported by empirical evidence. Research with elementary school staff highlights a desire for such programmes (Silveira-Zaldivar & Curtis, 2019). While many video modelling studies focus on younger children, positive outcomes are also observed in second-level students (Galligan et al., 2020). The Program for the Education and Enrichment of Relational Skills (PEERS) for high school students employs strategies such as didactic lessons and role-play demonstrations, showing ovrall improvements in social awareness and communication (Laugeson et al., 2012). Here, students navigate various social skills such as friendship maintenance, and handling conflict. Chang and Dean (2022) conducted a systematic review of school-based social skills interventions for autistic youth, revealing that these interventions were largely rooted in evidence-based practices and effectively enhanced social outcomes in inclusive educational settings. For instance, the ‘Remaking Recess’ programme improved peer relationships on the playground and classroom connections, reducing solitary play (Kasari et al., 2016; Shih et al., 2019).

### Game-based interventions

Play is crucial for child development, serving as a platform for learning social skills (Gresham, 1981; Vygotsky, 1978). Consequently, exclusion from, or a lack of social play can impact the development of social abilities, potentially leading anxiety and loneliness (Bauminger et al., 2003), underscoring the significance of fostering social skills. Despite challenges in social skills, many autistic adolescents enjoy socialising, with computer games and socialising being common favourite activities (Clark & Adams, 2020). This highlights a misconception that autistic children do not like socialising (Jaswal & Akhtar, 2018). Furthermore, video games can yield positive outcomes for autistic children (Jiménez-Muñoz et al., 2022), improving daily life skills such as hygiene (Kang & Chang, 2019), enhancing emotion recognition (Fridenson-Hayo et al., 2017) and boosting concentration and self-esteem (Jouen et al., 2017).

Games, as ‘world-building activities’ (Goffman, 1961, p. 27), are immersive and can motivate players, making them effective tools for educational interventions. Gamification can enhance motivation, learning outcomes and engagement (Dichev & Dicheva, 2017). In addition, games can facilitate the development of emotional competencies (Dell’Angela et al., 2020) and provide immediate and consistent feedback (M. Moore & Calvert, 2000). Serious games, coined in the 1970s, are digital games designed not only for entertainment but also to achieve additional goals (Caserman et al., 2020), for example, emotion regulation and communication skills (Bazoolnejad et al., 2022). These games aim to achieve objectives while maintaining an enjoyable player experience (Caserman et al., 2020). For example, Pokémon Go, a 2016 mobile game, promotes physical activity and has positive social effects (Wang, 2021). One promising application of serious games is in teaching social skills to autistic youth (Bernardini et al., 2014). Role-playing games (RPGs) can offer a safe space for autistic people to practice social interactions, associated with improved friendships and emotional well-being (Katō, 2019; Kilmer et al., 2023).

### Aims of current article

Social skills are complex and multifaceted, involving behaviours performed in social contexts (Cordier et al., 2015; Wolstencroft et al., 2018). McMahon et al. (2013) categorise social skills into various domains, including nonverbal (gestures, etc.), verbal (metaphors, etc.) and social interaction (conversation, etc.). Despite their importance, there is no consensus on the best approach to designing interventions. This article reviews past research using games as social skills interventions for autistic children and youth, examining their methods and outcomes.

Through a narrative synthesis, of a systematic sample of studies, we seek to establish:

1. What game-based approaches were taken in the teaching of social skills to autistic children and youth?
2. Which approaches to game-based social skills training demonstrate positive results?
3. Which social skills are targeted in game-based interventions – and why?
4. What views and beliefs informed the foundation of each study? Have the researchers named a specific psychological intervention approach?

## Methods

This systematic scoping review follows guidelines set out by Boland et al. (2017). The search strategy can be seen in Table 1.

**Table 1.** Search strategy.

Component one		Component two		Component three		Component four
Autism		Game*		Social communication		Adolescent
OR		OR		OR		OR
ASD		Serious games		Social skills		Children
OR		OR		OR		OR
Autism Spectrum Disorder	AND	Role-playing game	AND	Friendships	AND	Young adult
OR		OR		OR		OR
Asperger’s Syndrome		RPG		Social		Youth
OR		OR				
Autistic		TRPG				

The * indicates that we searched for game and games.

As shown in Table 2, the eligibility criteria for the studies were determined according to the Sample, Phenomenon of Interest, Design, Evaluation, Research type (SPIDER) model. As outlined by Cooke et al. (2012), SPIDER is best suited to qualitative and mixed-method reviews.

**Table 2.** Inclusion and exclusion criteria.

	Inclusion	Exclusion
(S) Sample	Empirical studies with autistic children and young people.	Studies with participants over 21.
(PI) Phenomenon of Interest	Empirical studies related to social skills training through games, for autistic people.	Studies focused on other areas of neurodiversity, for example, ADHD.
(D) Design	Any relevant qualitative, quantitative or mixed-methods paper that carries out social skills training with autistic people. There are no limitations on publication date.	Papers published in non-peer reviewed sources, case studies or theoretical papers.
(E) Evaluation	Empirical studies related to the teaching of social skills to autistic people.	No outcome measures related to social skills training.
(R) Research Type	Qualitative, quantitative or mixed method.	Duplicate papers and those not published in English.

**Electronic Databases:**

1. PsychInfo
2. EBSCO
3. Scopus
4. Web of Science
5. PubMed

These databases were chosen as they come from a range of publishers.

**Data extraction:**

*General information:* Authors, publication venue, research question, citation.

*Procedure:* Teaching, game design and evaluation procedures. Study design, outcome measurement, was there a control group.

*Participants:* Sample size, gender ratio, age, professional diagnosis.

*Findings:* Findings of the aforementioned research questions.

**Data management:** The studies identified through searches of electronic databases were imported and screened using Zotero, and the systematic review management software, Rayyan. The process is detailed in table 3.

**Table 3.** PRISMA figure.

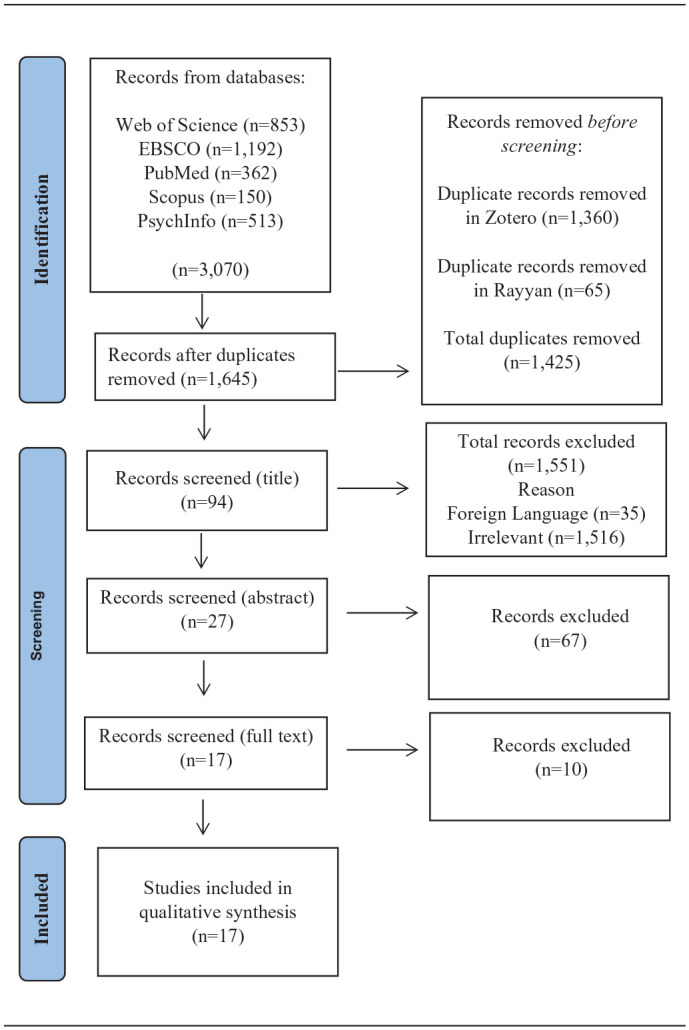

**Community Involvement and reflexivity:** There was no community involvement. I, the first author, am autistic. Thus, I am analysing the studies through a neurodivergent lens, and my own autistic experience. As an autistic person, I am personally engaged with the autistic community and their concerns regarding how research surrounding autism is conducted. Therefore, I made a conscious effort to maintain awareness of my involvement in the world I was studying.

**Appraisal tool:** We utilised the mixed methods appraisal tool (MMAT) (Hong et al., 2018). The MMAT is designed for the appraisal stage of systematic. Following MMAT guidelines, it is discouraged to calculate an overall score from the ratings of each criterion. It is also recommended against excluding studies of low methodological quality.

To ensure a comprehensive search strategy, we conducted a preliminary meeting with a librarian who provided guidance on developing search terms, selecting appropriate databases and refining the search strategy (Table 1).

As seen in Table 4, social skills encompass various facets of interaction and communication, both verbal and nonverbal. The term ‘communication’ was mentioned six times, while ‘interaction’, ‘collaboration’ and ‘attention’ each appeared three times. Social skills are often viewed as an individual’s effectiveness in engaging with others, considering factors like body language (Marchi et al., 2019; Tsai et al., 2020). Many behaviours are linked to social reciprocity, which assesses an individual’s willingness and ability to engage in reciprocal social interactions, including how they respond to others or negotiate in interpersonal situations (van Ommeren et al., 2011). Initiating social interactions and conveying thoughts and feelings are seen as important, as is expressing emotions through facial expressions and body language. Some studies explore specific aspects like metaphoric meaning (Barakova et al., 2009) and symbol use (Bernardini et al., 2014), emphasising the multifaceted nature of social skills. Overall, social skills can be categorised into social interaction, collaboration, joint attention and emotion expression, all of which are pertinent in understanding and supporting autistic people. Participants interacted with different partners depending on the study’s design. These partners include other participants, avatars or a combination of both. The number of sessions per participant varied widely across studies ranging from 1 to 31. For two (Bernardini et al., 2014; Silva et al., 2014), the number of sessions per individual participant was unclear.

**Table 4.** Number of participants, gender, age range, targeted social skills, who the participants were interacting with and the number of sessions per participant.

	No. of participants	Gender	Age range	Social skills	Who were the participants interacting with?	Number of sessions per participant?
Barakova et al., 2009	12	M: 11 F: 1	3–5	Social interaction and metaphoric meaning	Other participants	2
Bernardini et al., 2014	19	M: 18 F:1	4–14	Joint attention and symbol use	Avatar	Various times a week over 6 weeks
Chung et al., 2016	20	M: 17 F: 3	13–18	Social communication, facial recognition and emotional words	Other online players	18
Giusti et al., 2011	8	M: 8	9–12	Collaboration, joint performance, sharing, and mutual planning	Other participants	1
Guivarch et al., 2017	6	M: 4 F: 2	9–11	Co-operation and assertiveness	Other participants	22
Ioannou et al., 2020	77	M: 59 F: 18	8–16	Social competence and communication skills	Other participants	10
Jordan et al., 2013	3	N/A	19–21	Attention and communication	Other participants	3
Ke & Moon, 2018	8	M: 7 F: 1	10–14	Responding, initiation, interpersonal negotiation, positive self-identity expression, and cognitive flexibility	Avatars	16–31
Ke et al., 2022	7	M: 6 F: 1	10–14	Initiating social interactions, interpersonal negotiation, self-identity expression, and flexible thinking	Avatars	16–31
MacCormack & Freeman, 2019	4	M: 4	11–13	Self-competence and rates of initiation	Other participants	6
Mairena et al., 2019	15	M: 15	4–6	Social initiation conducts. Turn-taking, joint attention and vocalisation.	Other participants, adult and avatar	4
Marchi et al., 2019	10	M: 9 F: 1	8–11	Emotion expression and recognition. Expression of emotion via facial, vocal and bodily gestures	Avatars	10
Parsons, 2015	14	N/A	7–13	Social collaboration and perspective-taking	Other participants and avatar	3
Ribeiro et al., 2014	4	M: 3 F: 1	5–11	Overcoming challenges, development of communicative skills	Other participants and avatar	9
Silva et al., 2014	5	M: 3 F: 2	10–17	Collaboration, social interaction, communication, and sharing	Other participant	52 across 5 participants
Terlouw et al., 2021	37	M: 25 F: 12	10–12	Direct communication	Other participants	4
Tsai et al., 2020	3	M: 3	7–9	Social reciprocity behaviours	Avatar	10

**Table 5.** Game description, methods and outcome.

	Game	Social intervention	Outcome
Barakova et al., 2009	Multi-agent platform using interactive blocks	Games involving perceiving and understanding elements of social behaviour, including interactions between agents, and metaphoric meanings assigned to them	The use of an interactive multi-agent system (Game A) and adding metaphoric meanings (Game B) improved recognition of interaction patterns and metaphors, enhancing exploratory behaviour and social interactions
Bernardini et al., 2014	ECHOES	Interaction with an autonomous virtual agent in structured learning activities within a sensory garden environment	Using the ECHOES platform, which includes interactions with a virtual agent, improved rates of interaction and initiation
Chung et al., 2016	Poki-Poki	CBT	The use of a prosocial online game combined with CBT led to notable improvements in social interactions compared to traditional offline CBT
Giusti et al., 2011	Join-In Suite	CBT revolving around social stories emphasising specific collaboration dimensions	Effectiveness in enhancing collaboration, attention, negotiation and coordination stemmed from CBT-based interventions and specific game design elements like structured tasks and flexible phases
Guivarch et al., 2017	Various forms of adapted play (e.g., strategy games, board games, individual games)	Modification of game rules to promote communication and cooperation	Enhancements in adaptation, empathy, self-confidence and prosocial attitudes were linked to implicit social skills training and CBT-based interventions using structured group activities
Ioannou et al., 2020	SENSE Theatre	Theatrical activities such as mock auditions and imaginative play	Aspects of the intervention, such as peer-mediated play and imaginative activities, were linked to improvements in group play and reductions in trait anxiety
Jordan et al., 2013	Face Match	Personalised version of the game, played in three ways	Playing with a humanoid robot was associated with increased interaction, while repetitive behaviours were reduced
Ke & Moon, 2018	Open Simulator (3D virtual word)	Replication of various real-world settings and imaginative locales	The chess game was particularly effective for improving negotiation skills and cognitive flexibility, whereas role-playing simulation games were associated with a broad enhancement of social skills through varied game-based learning patterns
Ke et al., 2022	Open Simulator	Incorporation of play- and design-focused social interaction tasks within VR simulated everyday social scenarios	VR-based collaborative and design-oriented activities were most effective for enhancing negotiation, self-identity expression, and cognitive flexibility, whereas individual or competitive tasks often led to less predictable outcomes
MacCormack & Freeman, 2019	Minecraft	Structured play with specific roles and objectives and free play without	Structured play with specific roles and objectives was associated with improved rates of social initiations and the development of cooperative play dynamics, while free play showed gradual improvements in social skills but with less consistent and predictable outcomes
Mairena et al., 2019	Pico’s Adventure	Utilisation of Kinect sensor to capture child’s actions in a virtual environment with various tasks such as helping Pico fix its spaceship	The game led to significant increases in social initiation behaviours and a decrease in repetitive behaviours compared to free play, which showed less pronounced and consistent effects
Marchi et al., 2019	ASC-Inclusion Platform	A virtual environment themed as a research- camp in the jungle with animated characters and a smart reward system	Improvement in emotion and body language recognition, socialisation and gesture modality was achieved through a training programme that included two 15 min serious gaming sessions per week
Parsons, 2015	Block Challenge	Collaborative virtual reality environment	Teacher-led interventions, including direct communication guidance and conceptual explanations, significantly contributed to enhancing reciprocal and collaborative communication
Ribeiro et al., 2014	ComFiM	Multi-player environment for communication skill development	Improvements in communication skills were achieved through cooperative gameplay and a gradual reduction in therapist support
Silva et al., 2014	PAR	Collaboration using multitouch tabletops, changing pairs and roles in each game phase	Structured game phases and a mix of guided versus unrestricted interactions led to significant improvements in collaboration patterns and social interaction for users
Terlouw et al., 2021	AScapeD	Multi-player virtual escape room	The design of AScapeD facilitated increased collaboration, turn-taking, vocalisation and joint attention through balanced role distribution, interactive problem-solving tasks and feedback system that motivated players to work together
Tsai et al., 2020	Interactive 3D virtual reality RPG	Socialisation training within an immersive CAVE-like environment	The third-person perspective RPG system significantly enhanced autistic children’s ability to recognise and respond to emotions, facial expressions, and body language, resulting in improved role-play performance and sustained social skill development

## Narrative synthesis

### What game-based approaches were taken in the teaching of social skills to autistic children and youth?

Various game-based approaches were used to teach social skills. Some studies detailed their methodologies (Giusti et al., 2011; Marchi et al., 2019; Ribeiro et al., 2014; Silva et al., 2014; Terlouw et al., 2021; Tsai et al., 2020), while others focused on outcomes. This suggests a divergence in research methodologies, with some researchers prioritising the documentation of the game design process and approach, while others emphasise outcome evaluation.

Four interventions (Chung et al., 2016; Guivarch et al., 2017; Jordan et al., 2013; MacCormack & Freeman, 2019) used pre-existing games, including one adapted from a traditional matching pairs game (Jordan et al., 2013). The images on the cards depicted familiar individuals, drawing on autistic people’s preference for familiarity and predictability (Prizant et al., 2003).

Several studies developed new games (Barakova et al., 2009; Bernardini et al., 2014; Giusti et al., 2011; Mairena et al., 2019; Marchi et al., 2019; Parsons, 2015; Ribeiro et al., 2014; Silva et al., 2014; Terlouw et al., 2021; Tsai et al., 2020). For example, Terlouw et al. (2021) created an escape room game using the Design Research Framework for iterative development. This approach involved incremental enhancements to refine and enrich the escape room experience. Other games introduced unique characters for interaction (Bernardini et al., 2014; Mairena et al., 2019).

Virtual worlds through OpenSimulator were explored in two studies (Ke et al., 2022; Ke & Moon, 2018). Another study developed a 3D cave virtual environment RPG (Tsai et al., 2020). Participants, aged 7–9 and attending mainstream schools, engaged in the RPG, encountering various social scenarios. Multiple cameras were employed to enable players to view themselves from different perspectives, potentially fostering self-reflection as they observe their interactions. Elsewhere, cameras were used to capture facial, vocal and bodily expressions to understand emotional expression (Marchi et al., 2019).

Ioannou et al. (2020) improved social play skills through a theatre-based programme and at-home video modelling, a proven method for teaching various skills to autistic learners (Aldi et al., 2016). Video modelling is a teaching method that utilises video recordings and playback equipment to visually demonstrate the targeted behaviour or skill. (Franzone & Collet-Klingenberg, 2008).

Technology was a prevailing theme, ranging from screen-based to physical elements like humanoid robots. Some games immersed participants in virtual worlds (Ke et al., 2022; Ke & Moon, 2018), while others integrated technology into real-life settings (Barakova et al., 2009). As noted, there was a discrepancy in how ‘social’ these social skills games were. For instance, some games (Bernardini et al., 2014; Mairena et al., 2019) involved children interacting or socialising with video game characters, while others required interaction with other children or adults (e.g. Parsons, 2015).

### Which approaches to game-based social skills training demonstrate positive results?

Two games were played from participants’ homes (Ke et al., 2022; Ke & Moon, 2018), while the others took place in various locations, including hospitals, primary schools and universities. Some used existing games for social skills intervention (Chung et al., 2016; Guivarch et al., 2017; Jordan et al., 2013; MacCormack & Freeman, 2019), while the others were designed specifically as social skills interventions. All interventions featured extensive adult facilitation. Thirteen of the 17 studies adhered to a manualised approach, which involved detailed, standardised guidelines for the delivery of the intervention. However, there were exceptions where the interventions were not strictly manualised (Barakova et al., 2009; Bernardini et al., 2014; Guivarch et al., 2017; Ribeiro et al., 2014). For instance, Bernardini et al. (2014) used a flexible, adaptive approach in the ECHOES intervention.

As outlined in table 6, serious games like ECHOES often use virtual agents or environments to create immersive experiences for enhancing social skills. Tabletop games, including both co-located and multitouch variants, introduce a tangible and collaborative dimension to skill development. Co-located games rely on physical components like boards and cards, while multitouch variants use digital displays and touch inputs. RPGs offer engaging settings for skill development. While serious games, virtual environments and virtual reality games are common, the prevalence of serious games may stem from their versatility in addressing various therapeutic goals The choice of game type likely depends on the specific social skill objectives.

**Table 6.** Types of games.

**Types of games**		
A multi-agent platform using interactive blocks.		A virtual reality based social skills learning environment.
The serious game, ECHOES, which includes an autonomous virtual agent.		A 3D virtual playground.
Prosocial online game.		Picos adventure, utilising a Kinect sensor.
A collection of co-located games on a tabletop device.		The ASC-Inclusion platform serious virtual game.
Various forms of play, such as strategy games, board games.		A collaborative virtual reality environment.
SENSE Theatre.		ComFiM, a game utilising two tablets.
The game face match was played using cards, a smart board and a robot.		PAR is a multi-user collaborative game tailored for multitouch tabletops.
Minecraft.		Virtual escape room. CAVE-like immersive 3D virtual reality role-playing game.

All studies showed that game-based interventions promoted social skills in autistic children and youth, using various outcome measures. For example, Ke et al. (2022) reported improvements in negotiation, self-identity expression and cognitive flexibility in 10–14-year-old participants after a 20 h virtual reality programme. Enhanced self-identity expression included expressing preferences and explaining personal perspectives. High-pressure social scenarios like job interviews posed challenges to self-identity expression. Ioannou et al. (2020) found that theatrical performance reduced self-reported anxiety and fostered social engagement. However, some studies noted limitations; for instance, Guivarch et al. (2017) saw significant improvements in certain areas but not others, such as anxiety. Different definitions of social skills across studies complicate direct comparisons.

The game ECHOES was developed based on the Social Communication, Emotional Regulation, and Transactional Support (SCERTS) framework, specifically tailored for autistic children (Bernardini et al., 2014) and emphasises an individualised, family-centred approach to intervention (Prizant et al., 2006). ECHOES features a virtual character named Andy, who greets the child by name and offers encouraging feedback. Affirmative feedback has been identified as a motivator for children and youth (de Haas et al., 2020). While this can motivate children, it raises questions about the ‘social’ nature of interactions with a video game character. These interactions may be parasocial, giving an illusion of reciprocity but lacking real-life social nuances. Thus, such games should complement, not replace, other social skills development methods (Zheng et al., 2021).

There was a discrepancy in the rigour of outcome measurement methods. Some studies used pre- and post-measures with observation checklists, while others relied solely on observations. In studies with less robust methodologies, observed improvements may be influenced by other factors. For instance, Barakova et al. (2009) compared two games and found more social interactions in the game with metaphorical meaning, but the lack of pre- and post-testing makes it hard to attribute differences solely to the intervention. Comprehensive outcome measures, including both pre- and post-assessments, would enhance the robustness of findings and better attribute improvements to the interventions.

This limitation is evident in several studies. Without pre- and post-measures, discerning the specific impact of game-based interventions is compromised. Including comprehensive outcome measures with both pre- and post-assessments would enhance the robustness of findings and allow researchers to attribute differences more confidently to the intervention.

### Which social skills are targeted in game-based interventions – and why?

Across the studies, there was a lack of specificity in defining and discussing social skills, with many failing to justify why particular skills were chosen for intervention. Instead, they broadly referred to current diagnostic criteria. While it is logical that interventions may include some form of social communication training, this could encompass various elements (Newschaffer et al., 2007), including verbal and nonverbal factors. Moreover, within each diagnostic category, multiple domains of function have influence and impact. Thus, solely referring to the broad characteristics of autistic people does not adequately justify why specific skills, such as collaboration (Silva et al., 2014) or turn-taking (Mairena et al., 2019), are targeted for intervention.

The concept of social reciprocity, the capacity to engage in social interactions involving two or more people (Schwartz et al., 2021), was evident in several studies. In addition, it is defined as the ability to process social information, understand spoken communication and appropriately respond in interpersonal interactions (Constantino et al., 2000). As seen in Table 7, although not all explicitly used the term, the targeted behaviours often aligned with its definition.

**Table 7.** Socially reciprocal behaviours.

Authors	Social reciprocity
Bernardini et al., 2014; Terlouw et al., 2021	Turn-taking and equal co-operation
Giusti et al., 2011	Joint performance. Performance of an action together
Giusti et al., 2011; Parsons, 2015; Ribeiro et al., 2014	Collaboration. Mutual planning and sharing
Ke & Moon, 2018; Ke et al., 2022	Negotiation. For example, developing a shared goal for gameplay
MacCormack & Freeman, 2019; Mairena et al., 2019	The initiation of direct communication with peers and rates of initiation

Not all social skills fall under social reciprocity. Other studies explored specific skills like understanding others’ perspectives (Ke et al., 2022; Ke & Moon, 2018; Parsons, 2015; Tsai et al., 2020), cognitive flexibility (Ke et al., 2022; Ke & Moon, 2018) and learning new vocabulary (Ribeiro et al., 2014). These skills do not necessarily rely on reciprocal interactions.

Some studies provided clear rationales for selecting specific skills (Barakova et al., 2009; Bernardini et al., 2014; Ioannou et al., 2020; Ke & Moon, 2018; MacCormack & Freeman, 2019; Terlouw et al., 2021; Tsai et al., 2020). For example, Terlouw et al. (2021) aimed to facilitate direct communication between autistic children and peers, citing conversational challenges. MacCormack and Freeman (2019) focused on social competence to address the negative outcomes of social exclusion. However, while justifying their focus on social competence, the latter study lacks a comprehensive description of its components, leaving room for interpretation.

Often, the rationale for selecting specific skills and their benefits for autistic people was unclear (Chung et al., 2016; Giusti et al., 2011; Jordan et al., 2013; Ke et al., 2022; Marchi et al., 2019; Parsons, 2015; Ribeiro et al., 2014; Silva et al., 2014). For example, Mairena et al. (2019) targeted social initiation skills, such as achieving eye contact and joint attention, but did not clearly explain their relevance to autism. Is eye contact crucial for social initiation, or is it merely perceived as important by neurotypical people? This ambiguity persists across our sample of studies. Thus, it is crucial to ask: Why are these the skills being taught? Are we training autistic people to conform to neurotypical standards, or are we enhancing their quality of life?

Despite inconsistencies, authors could have established a taxonomy of skills and their definitions and provided an overview of the most and least targeted skills. Without clear definitions and descriptions of how games address these skills, it is challenging to conclusively assert their improvement. The absence of a clear rationale for selecting these skills raises doubts about whether these interventions prioritise the best interests of autistic people.

### What views and beliefs informed the foundation of each study? Have the researchers named a specific psychological intervention approach?

These questions were asked to understand the motivations behind each study and the literature that informed it. There were two headings that our sample of studies could fit under.

Studies that focused on knowledge of technology, and past research regarding technological interventions (Table 8).
Studies that focused on knowledge of autism, and past research on SST and autism (Table 9).

Many studies were based on past research regarding technology. Utilising technology in these interventions seems logical, given its well-researched benefits for autistic people.

For example, computer-aided learning (D. Moore et al., 2000). In addition, autistic people often have a strong inclination towards gaming, particularly video games (Clark & Adams, 2020; Kapp, 2012). However, the prevalent use of technology raises the question: Could the perceived advantages of technology have overshadowed the need for a clearer definition of social skills in these studies?

**Table 8.** How past technological research informed the studies theoretical approach.

Authors	Technology research
Bernardini et al., 2014; Ke et al., 2022; Terlouw et al., 2021	Past studies show that many autistic people exhibit a natural affinity for technology and a positive attitude towards computer-based training. These spaces may offer a predictable and structured environment, which can accommodate for a preference for routine or repetitive behaviours.
Ke & Moon, 2018	VR can provide a space for naturalistic social skills training. It has an intrinsic appeal to visual learners. The non-threatening environment that VR can cultivate allows users to safely practise social skills.
Parsons, 2015	Technology-enhanced learning and educational technologies can be designed for supporting learning in core areas of difficulty.
Ribeiro et al., 2014	One of the benefits of technological interventions is the flexibility they can offer. For example, the environment can be customised according to specific needs.
Tsai et al., 2020	Video-modelling can help autistic children to understand/reflect on social behaviours. It can also assist them in understanding the perspective of others and how their behaviour impacts them.

**Table 9.** How past autism research informed the studies theoretical approach.

Authors	Technology research
Bernardini et al., 2014	Activity-based interventions might be preferable for autistic people as they provide structure or a concrete focal point.
Ioannou et al., 2020	This study looked at theatrical role-play and research on autism and social anxiety.
Ke & Moon, 2018	A social skills programme should enhance the autistic person’s attention and motivation towards social information and interaction. This can be achieved through increasing the relevance of the social stimulus in solving a task.
MacCormack & Freeman, 2019	When autistic youth are left out from play, they do not get the opportunities they need to practise or develop social abilities. This can result in the young person feeling isolated. Peer mediation can help autistic youth improve their social competence.
Silva et al., 2014	Collaborative patterns were focused on as they help to encourage collaboration for autistic people. Such contribute to cooperative skills and the generation of social interactions.

#### Have the researchers named a specific autism-approach in relation to the intervention?

Regarding the intervention approaches, some studies did not follow a specific approach (Jordan et al., 2013; Ke et al., 2022; Marchi et al., 2019). Two studies adhered to cognitive behavioural therapy principles (Chung et al., 2016; Silva et al., 2014). Others followed pre-existing frameworks (Bernardini et al., 2014; Ribeiro et al., 2014; Terlouw et al., 2021), while Guivarch et al. (2017) focused on the implicit modality (bottom-up). Additional approaches included third-person perspective training (Tsai et al., 2020), robots and behavioural training (Barakova et al., 2009), naturalistic interventions (Ke & Moon, 2018), observational methods (Ioannou et al., 2020; MacCormack & Freeman, 2019; Mairena et al., 2019), models of learner-centred design (Parsons, 2015) and collaborative patterns (Silva et al., 2014).

## Discussion

Our synthesis reveals a diverse range of game-based approaches for teaching social skills to autistic children and youth. These approaches vary significantly in their design and objectives, reflecting different strategies for achieving SST outcomes. For instance, serious games like ECHOES emphasise individualised, family-centred interventions (Bernardini et al., 2014), while others incorporate cognitive behavioural therapy principles to enhance social interaction skills (Chung et al., 2016; Silva et al., 2014). This diversity underscores the importance of aligning game design with specific social skill objectives to ensure effectiveness. Social interaction level varied, from virtual character interactions (e.g. Bernardini et al., 2014) to real-life engagements (e.g. Parsons, 2015), suggesting further research is needed to balance technological and real-world interactions for effective SST.

Our review shows that game-based interventions are generally effective in improving participant performance on measures of social skills. For example, children recognised metaphors presented through embodied agents (Barakova et al., 2009), and a virtual reality environment improved negotiation, self-identity expression and cognitive flexibility (Ke et al., 2022). However, there is a discrepancy in the rigour of outcome measurement methods. To effectively evaluate and advance these interventions, comprehensive outcome measures, including pre- and post-assessments, are crucial for determining their impact and refining future strategies.

Our synthesis reveals a lack of specificity in defining and justifying chosen skills. While social reciprocity was a common focus, many studies did not clearly explain why specific skills, such as collaboration or turn-taking, were selected (Mairena et al., 2019; Silva et al., 2014). Although some studies did provide clear rationales (e.g. Terlouw et al., 2021), there was often ambiguity about the relevance of these skills to the autistic experience. For instance, targeting eye contact might reflect neurotypical expectations rather than addressing autistic needs. The lack of a detailed rationale complicates the evaluation of these interventions’ effectiveness.

The foundations of studies were informed by two primary areas: knowledge of technology or understanding of autism. Many studies focused on the benefits of computer-aided learning, raising questions about whether technological benefits overshadow the need for a clear definition of social skills. The approaches varied from cognitive behavioural therapy principles (Chung et al., 2016; Silva et al., 2014) to frameworks like SCERTS (Bernardini et al., 2014). The diversity in foundational beliefs and the lack of a consistent approach highlights the need for a framework that considers both technological benefits and the specific social skill needs of autistic people.

Finally, our review highlights several shortcomings of past studies, identifying areas for improvement and future work. Several studies noted the necessity for further research to enhance generalisation (Chung et al., 2016; Ioannou et al., 2020; Ke et al., 2022; Mairena et al., 2019). While virtual reality (Ke & Moon, 2018) and virtual agents (Bernardini et al., 2014) show promise, future work should assess how well skills transfer from games to real-life contexts. Some games, for example, Giusti et al. (2011), necessitate collaborative play for success. While in-game enforcement mechanisms ensure adherence to rules, such structures may not exist outside of the gaming context. In game design, many studies missed opportunities to include autistic people’s perspectives on social skills, focusing instead on game mechanics (e.g. Ke et al., 2022). We recommend including autistic youth and adults in collaborative research to adhere to the principle of ‘nothing about us without us’ (Poulsen et al., 2022). Existing literature often defines social skills from a neurotypical perspective, risking stigmatisation and stereotyping. Inclusive research must prioritise autistic voices for a more accurate understanding of social skills.

This study contributes to the literature on game design for SST by consolidating empirical studies and offering insights into various approaches and their successes. However, past research has left many gaps, for example, there are game like processes that have not been captured. As the literature largely focused on games designed for use in a therapeutic setting, school-based games may have been missed. In addition, it is conceivable that innovative gaming technologies have yet to be explored.

## Supplemental Material

sj-odt-1-aut-10.1177_13623613241277309 – Supplemental material for Is there evidence that playing games promotes social skills training for autistic children and youth? Supplemental material, sj-odt-1-aut-10.1177_13623613241277309 for Is there evidence that playing games promotes social skills training for autistic children and youth? by Orla Walsh, Conor Linehan and Christian Ryan in Autism
