# Supplementary material for: Is there evidence that playing games promotes social skills training for autistic children and youth?
Source: Autism. 2024 Sep 21;29(2):329–43. doi: 10.1177/13623613241277309 (PMC11816463; doi:10.1177/13623613241277309)
Supplement: Supplementary file 1 [file sj-odt-1-aut-10.1177_13623613241277309.odt]

Appendix One

Mixed Methods

Barakova et al., 2009

|  | Answers |
| --- | --- |
| Are there clear research questions? | Yes. |
| Do the collected data allow to address the research questions? | Yes. |
| Is there an adequate rationale for using a mixed methods design to address the research question? | No. |
| Are the different components of the study effectively integrated to answer the research question? | Yes. |
| Are the outputs of the integration of qualitative and quantitative components adequately interpreted? | Yes. |
| Are divergences and inconsistencies between quantitative and qualitative results adequately addressed? | No. |
| Do the different components of the study adhere to the quality criteria of each tradition of the methods involved? | Yes. |

Mixed Methods

Bernardini et al., 2014

|  | Answers |
| --- | --- |
| Are there clear research questions? | Yes. |
| Do the collected data allow to address the research questions? | Yes. |
| Is there an adequate rationale for using a mixed methods design to address the research question? | Yes. |
| Are the different components of the study effectively integrated to answer the research question? | Yes. |
| Are the outputs of the integration of qualitative and quantitative components adequately interpreted? | Yes. |
| Are divergences and inconsistencies between quantitative and qualitative results adequately addressed? | No. |
| Do the different components of the study adhere to the quality criteria of each tradition of the methods involved? | Can’t tell. |

Mixed Methods

Chung et al., 2016

|  | Answers |
| --- | --- |
| Are there clear research questions? | Yes. |
| Do the collected data allow to address the research questions? | Yes. |
| Is there an adequate rationale for using a mixed methods design to address the research question? | Yes. |
| Are the different components of the study effectively integrated to answer the research question? | Yes. |
| Are the outputs of the integration of qualitative and quantitative components adequately interpreted? | Yes. |
| Are divergences and inconsistencies between quantitative and qualitative results adequately addressed? | No. |
| Do the different components of the study adhere to the quality criteria of each tradition of the methods involved? | Can’t tell. |

Qualitative

Giusti et al., 2011

|  | Answers |
| --- | --- |
| Are there clear research questions? | Yes. |
| Do the collected data allow to address the research questions? | Yes. |
| Is the qualitative approach appropriate to answer the research question? | Yes |
| Are the qualitative data collection methods adequate to address the research question? | Yes. |
| Are the findings adequately derived from the data? | Yes. |
| Is the interpretation of results sufficiently substantiated by data? | Yes. |
| Is there coherence between qualitative data sources, collection, analysis, and interpretation? | Can’t tell. |

Quantitative Descriptive

Guivarch et al., 2017

|  | Answers |
| --- | --- |
| Are there clear research questions? | Yes. |
| Do the collected data allow to address the research questions? | Yes. |
| Is the sampling strategy relevant to address the research question? | Yes. |
| Is the sample representative of the target population? | Can’t tell. |
| Are the measurements appropriate? | Yes. |
| Is the risk of nonresponse bias low? | Can’t tell. |
| Is the statistical analysis appropriate to answer the research question? | Yes. |

Mixed Methods

Ioannou et al., 2020

|  | Answers |
| --- | --- |
| Are there clear research questions? | Yes. |
| Do the collected data allow to address the research questions? | Yes. |
| Is there an adequate rationale for using a mixed methods design to address the research question? | No. |
| Are the different components of the study effectively integrated to answer the research question? | Yes. |
| Are the outputs of the integration of qualitative and quantitative components adequately interpreted? | No. |
| Are divergences and inconsistencies between quantitative and qualitative results adequately addressed? | No. |
| Do the different components of the study adhere to the quality criteria of each tradition of the methods involved? | Can’t tell. |

Mixed Methods

Jordan et al., 2013

|  | Answers |
| --- | --- |
| Are there clear research questions? | Yes. |
| Do the collected data allow to address the research questions? | Yes. |
| Is there an adequate rationale for using a mixed methods design to address the research question? | Yes. |
| Are the different components of the study effectively integrated to answer the research question? | Yes. |
| Are the outputs of the integration of qualitative and quantitative components adequately interpreted? | Yes. |
| Are divergences and inconsistencies between quantitative and qualitative results adequately addressed? | Yes. |
| Do the different components of the study adhere to the quality criteria of each tradition of the methods involved? | Can’t tell. |

Mixed Methods

Ke & Moon, 2018

|  | Answers |
| --- | --- |
| Are there clear research questions? | Yes. |
| Do the collected data allow to address the research questions? | Yes. |
| Is there an adequate rationale for using a mixed methods design to address the research question? | Yes. |
| Are the different components of the study effectively integrated to answer the research question? | Yes. |
| Are the outputs of the integration of qualitative and quantitative components adequately interpreted? | Yes. |
| Are divergences and inconsistencies between quantitative and qualitative results adequately addressed? | No. |
| Do the different components of the study adhere to the quality criteria of each tradition of the methods involved? | Yes. |

Mixed Methods

Ke et al., 2022

|  | Answers |
| --- | --- |
| Are there clear research questions? | Yes. |
| Do the collected data allow to address the research questions? | Yes. |
| Is there an adequate rationale for using a mixed methods design to address the research question? | No. |
| Are the different components of the study effectively integrated to answer the research question? | Yes. |
| Are the outputs of the integration of qualitative and quantitative components adequately interpreted? | Yes. |
| Are divergences and inconsistencies between quantitative and qualitative results adequately addressed? | No. |
| Do the different components of the study adhere to the quality criteria of each tradition of the methods involved? | Yes. |

Mixed Methods

MacCormack & Freeman, 2019

|  | Answers |
| --- | --- |
| Are there clear research questions? | Yes. |
| Do the collected data allow to address the research questions? | Yes. |
| Is there an adequate rationale for using a mixed methods design to address the research question? | No. |
| Are the different components of the study effectively integrated to answer the research question? | Yes. |
| Are the outputs of the integration of qualitative and quantitative components adequately interpreted? | Yes. |
| Are divergences and inconsistencies between quantitative and qualitative results adequately addressed? | No. |
| Do the different components of the study adhere to the quality criteria of each tradition of the methods involved? | No. |

Mixed Methods

Mairena et al., 2019

|  | Answers |
| --- | --- |
| Are there clear research questions? | Yes. |
| Do the collected data allow to address the research questions? | Yes. |
| Is there an adequate rationale for using a mixed methods design to address the research question? | Yes. |
| Are the different components of the study effectively integrated to answer the research question? | Yes. |
| Are the outputs of the integration of qualitative and quantitative components adequately interpreted? | Yes. |
| Are divergences and inconsistencies between quantitative and qualitative results adequately addressed? | No. |
| Do the different components of the study adhere to the quality criteria of each tradition of the methods involved? | Yes. |

Mixed Methods

Marchi et al., 2019

|  | Answers |
| --- | --- |
| Are there clear research questions? | Yes. |
| Do the collected data allow to address the research questions? | Yes. |
| Is there an adequate rationale for using a mixed methods design to address the research question? | Yes. |
| Are the different components of the study effectively integrated to answer the research question? | Yes. |
| Are the outputs of the integration of qualitative and quantitative components adequately interpreted? | No. |
| Are divergences and inconsistencies between quantitative and qualitative results adequately addressed? | No. |
| Do the different components of the study adhere to the quality criteria of each tradition of the methods involved? | Can’t tell. |

Mixed Methods

Parsons, 2015

|  | Answers |
| --- | --- |
| Are there clear research questions? | Yes. |
| Do the collected data allow to address the research questions? | Yes. |
| Is there an adequate rationale for using a mixed methods design to address the research question? | Yes. |
| Are the different components of the study effectively integrated to answer the research question? | No. |
| Are the outputs of the integration of qualitative and quantitative components adequately interpreted? | Yes. |
| Are divergences and inconsistencies between quantitative and qualitative results adequately addressed? | No. |
| Do the different components of the study adhere to the quality criteria of each tradition of the methods involved? | Yes. |

Mixed methods

Ribeiro et al., 2014

|  | Answers |
| --- | --- |
| Are there clear research questions? | Yes. |
| Do the collected data allow to address the research questions? | Yes. |
| Is there an adequate rationale for using a mixed methods design to address the research question? | Yes. |
| Are the different components of the study effectively integrated to answer the research question? | Yes. |
| Are the outputs of the integration of qualitative and quantitative components adequately interpreted? | Yes. |
| Are divergences and inconsistencies between quantitative and qualitative results adequately addressed? | No. |
| Do the different components of the study adhere to the quality criteria of each tradition of the methods involved? | Can’t tell. |

Mixed Methods

Silva et al., 2014

|  | Answers |
| --- | --- |
| Are there clear research questions? | Yes. |
| Do the collected data allow to address the research questions? | Yes. |
| Is there an adequate rationale for using a mixed methods design to address the research question? | No. |
| Are the different components of the study effectively integrated to answer the research question? | Can’t tell. |
| Are the outputs of the integration of qualitative and quantitative components adequately interpreted? | Yes. |
| Are divergences and inconsistencies between quantitative and qualitative results adequately addressed? | No. |
| Do the different components of the study adhere to the quality criteria of each tradition of the methods involved? | Yes. |

Mixed Methods

Terlouw et al., 2017

|  | Answers |
| --- | --- |
| Are there clear research questions? | Yes |
| Do the collected data allow to address the research questions? | Yes |
| Is there an adequate rationale for using a mixed methods design to address the research question? | Yes |
| Are the different components of the study effectively integrated to answer the research question? | Yes |
| Are the outputs of the integration of qualitative and quantitative components adequately interpreted? | Yes |
| Are divergences and inconsistencies between quantitative and qualitative results adequately addressed? | Yes |
| Do the different components of the study adhere to the quality criteria of each tradition of the methods involved? | Yes |

Mixed methods

Tsai et al., 2021

|  | Answers |
| --- | --- |
| Are there clear research questions? | Yes |
| Do the collected data allow to address the research questions? | Yes |
| Is there an adequate rationale for using a mixed methods design to address the research question? | Yes |
| Are the different components of the study effectively integrated to answer the research question? | Yes |
| Are the outputs of the integration of qualitative and quantitative components adequately interpreted? | Yes |
| Are divergences and inconsistencies between quantitative and qualitative results adequately addressed? | No |
| Do the different components of the study adhere to the quality criteria of each tradition of the methods involved? | Yes |
